# Pheochromocytoma disguised as gestational hypertensive disease during pregnancy: A case report

**DOI:** 10.1002/ccr3.6932

**Published:** 2023-02-10

**Authors:** Swati Kumari, Ramesh Lamichhane, Subarna Giri, Saroj Chaudhary, Shanta Neupane, Neha Dangol

**Affiliations:** ^1^ Department of Gynecology and Obstetrics Tribhuvan University Institute of Medicine Kathmandu Nepal; ^2^ Nepal Cardio Diabetes and Thyroid Center Kathmandu Nepal; ^3^ Maharajgunj Medical Campus Tribhuvan University Institute of Medicine Kathmandu Nepal

**Keywords:** gynecologic oncology, hypertension, pheochromocytoma, pregnancy

## Abstract

Pheochromocytoma is often diagnosed prior to pregnancy. Sometimes, the disease may be diagnosed for the first‐time during pregnancy masking itself as a hypertensive disease in pregnancy. Early diagnosis and timely, appropriate management reduce possible maternal and fetal complications. We identified a case of pheochromocytoma during pregnancy misdiagnosed as preeclampsia.

## Graphical abstract

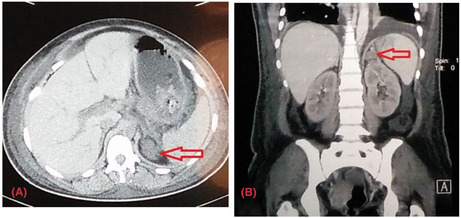

## 1 INTRODUCTION

Pheochromocytoma is a catecholamine‐secreting tumor that is rare during pregnancy, with a prevalence of <0.2 per 10,000 pregnancies^1^ Recognizing pheochromocytoma antenatally is difficult because it may mimic gestational hypertensive diseases. Maternal and fetal mortality rates are high in case there is a late diagnosis.^2^ In 20% of the cases, diagnosis is not made during pregnancy.^3^ Early diagnosis and treatment decrease maternal and fetal death rates to less than 5% and 15%, respectively^4^.

We report the case of a patient with pheochromocytoma that was diagnosed during postpartum and the complexity of its management.

## 2 CASE PRESENTATION

A 30‐year‐old primipara lady was admitted to our hospital following an emergency lower segment cesarean section at 37 + 2 weeks for severe preeclampsia on her fourth postpartum day. She was referred for the management of uncontrolled hypertension and vomiting for 4 days.

She had been initially diagnosed with systemic hypertension (Blood pressure = 180/100 mm Hg) at the 8th week at a local health post on her first antenatal visit. She was managed with oral amlodipine 5 mg (stat then once daily) and methyldopa (500 mg, TDS), which she had taken throughout her pregnancy. The antenatal period was otherwise uneventful until the patient developed a BP of 220/120 mm Hg at 37 weeks of gestation. In addition, she had a headache, and multiple episodes of vomiting, and her BP was persistently high in spite of maximum doses of antihypertensive medication (IV labetalol + IV magnesium sulphate). Due to failure to progress cervical change for 4 h despite adequate contraction, she underwent an emergency lower segment cesarean section. Her provisional diagnosis was severe preeclampsia superimposed on chronic hypertension. Cesarean section was uneventful and intraoperative fluctuations in BP were managed with nitroprusside. She gave birth to a 3.1 kg female with APGAR scores of 8/10, and 9/10 at 1 and 5 min, respectively. There were no neonatal complications. During the postpartum period, the blood pressure remained persistently high, and ultimately the patient was referred to our center for further management.

On presentation to our hospital, the general condition was fair. There were no pallor, icterus, lymphadenopathy, and dehydration. Bilateral pedal edema was present. The temperature was 99 ° F, BP 230/130 mm Hg bilaterally, pulse 120 beats/min and regular, and saturation 92% in room air. Her breasts and thyroid examination were normal. Systemic examination showed that the abdomen was distended and tender with normal bowel sound. Moreover, the fluid thrill was positive. Also, the respiratory examination demonstrated decreased air entry in the bilateral basal region.

**TABLE 1:** Comparing the differentiating point of pheochromocytoma and preeclampsia with our case

	Preeclampsia	Pheochromocytoma	Our case
Clinical features			
Time of presentation	>20 weeks of gestation	Variable (any time during pregnancy)	8th week of gestation
Hypertension	Sustained	Paroxysmal (almost 45%) Sustained (Almost half) Normotensive (5%–15%)	Sustained
Bipedal edema	Usually present	Absent	3+ edema present
Orthostatic hypotension	Absent	Present (40%)	Absent
Laboratory markers			
Proteinuria	24 h urine collection: ≥300 mg/24 h	Absent	2 gm./24 h
Catecholamines	Normal	Elevated	Elevated
Glucose	Normal	Hyperglycemic (50% of the patients)	Normal (82 mg/dL)

Hematologic investigation revealed slightly low hemoglobin with significant leukocytosis. The biochemistry result showed low potassium. Other parameters were normal including 24 h urinary pheochromocytoma evaluation (epinephrine, norepinephrine, normetanephrine, and vanillylmandelic acid) (Table 2).

**TABLE 2:** Laboratory markers of our patient

Tests	Preoperative	Postoperative	Reference range
Hematological and biochemical			
Hemoglobin	10.9	10.7	12–15 g/dL
Serum creatinine	0.7	1.1	0.5–0.9 mg/dL
Sodium	142	143	135–145 meq/L
Chloride	102	100	96–106 mmol/L
Potassium	3.7	2.5	3.6–5.2 mmol/L
Pheochromocytoma evaluation: 24 h urinary			
Vanillylmandelic acid	90	25	0–35 mcmol/24 h
Epinephrine	170	100	15–80 mcg/24 h
Norepinephrine	5521	500	<900 μg/day
Normetanephrines	17,000	1860	50–650 μg/day

**FIGURE 1:**
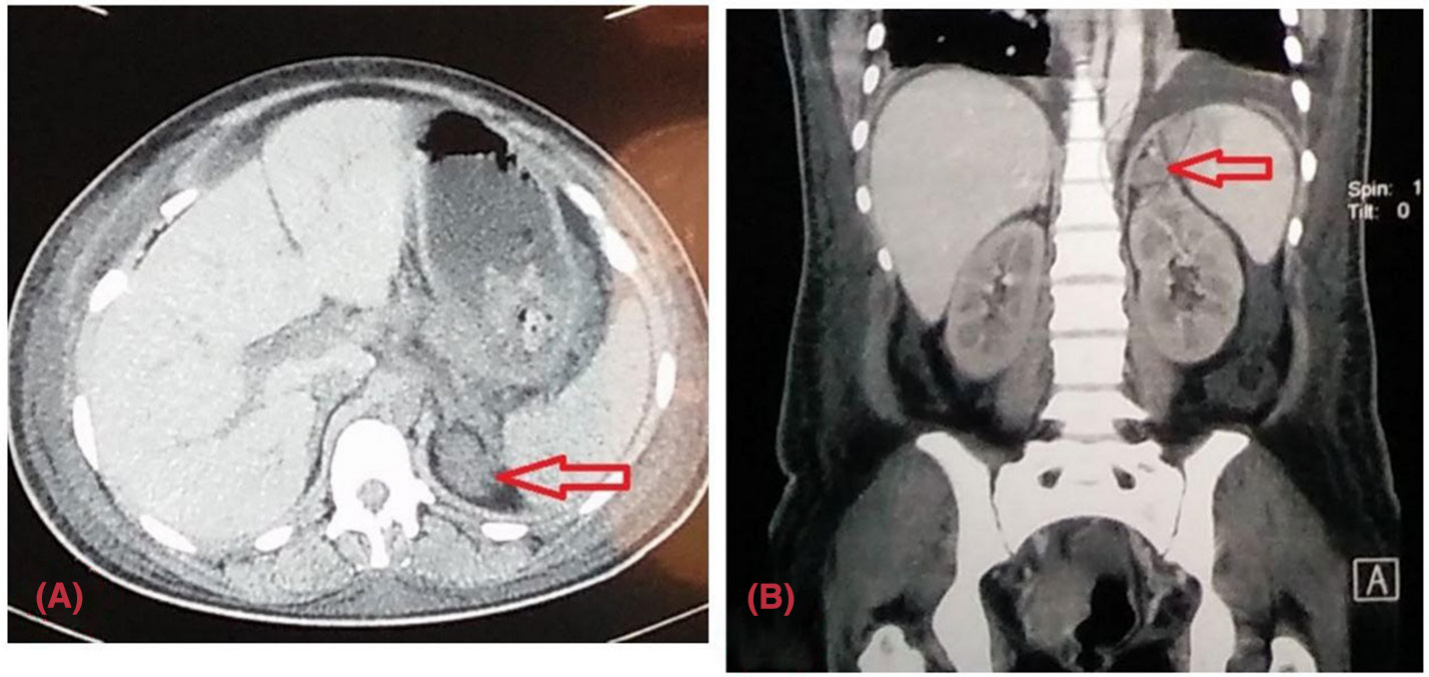
(A) CT coronal view showing supra renal mass (B) CT axial view showing adrenal mass

Ultrasonography revealed hepatomegaly, bulky postpartum uterus, gross ascites, and bilateral pleural effusion. CT scan of the abdominal region showed a 2.5 cm × 2 cm well‐defined hypodense lesion noted in the left adrenal gland (Figure 1). Echocardiography showed moderate concentric left ventricular hypertrophy, grade I left ventricular diastolic dysfunction, mild aortic regurgitation, minimal pericardial effusion, and an ejection fraction of 60%. The markers for pheochromocytoma were elevated as shown in Table 2. A provisional diagnosis of hypertensive crisis with hypokalemia with left adrenal adenoma was made.

**FIGURE 2:**
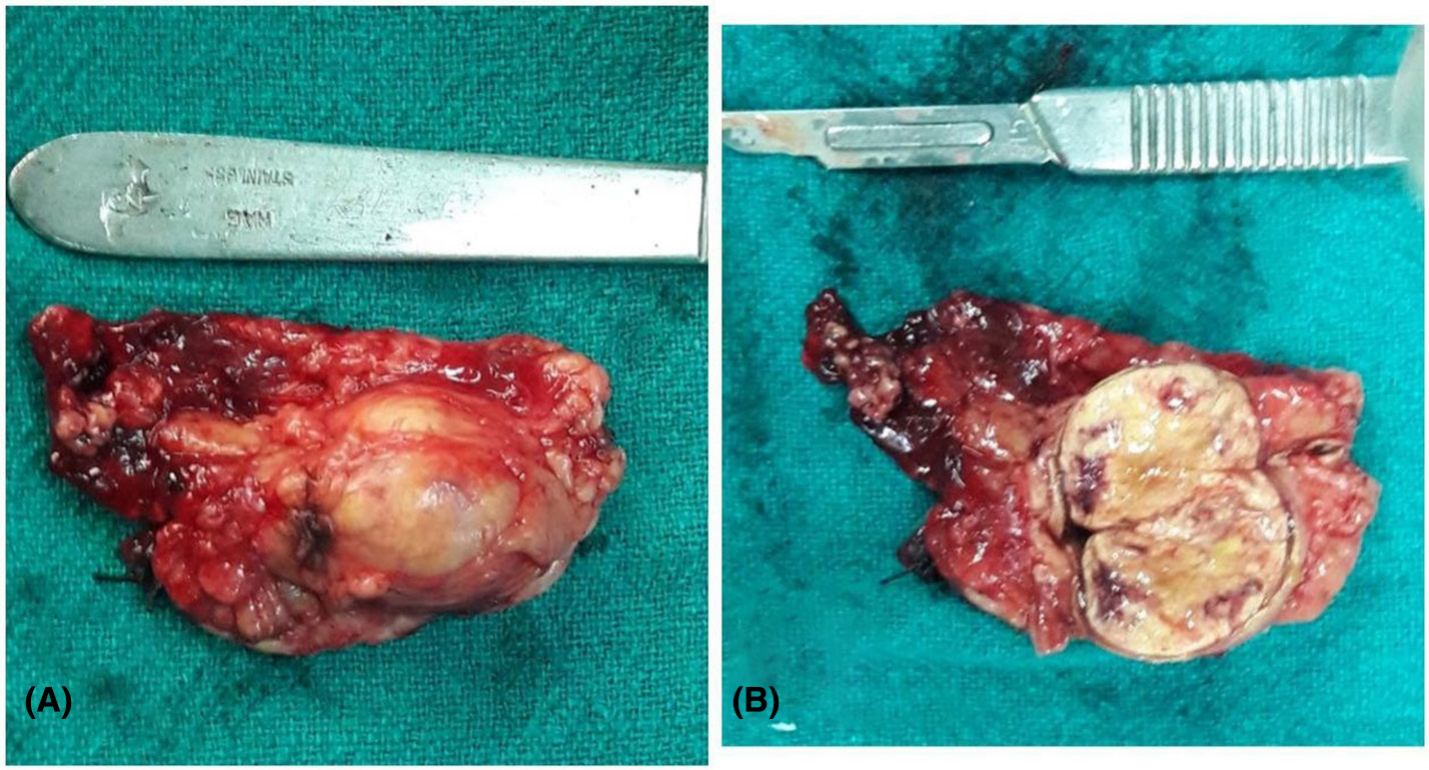
Showing resected specimen of adrenal gland (A) Gross view and (B) Cut section

**FIGURE 3:**
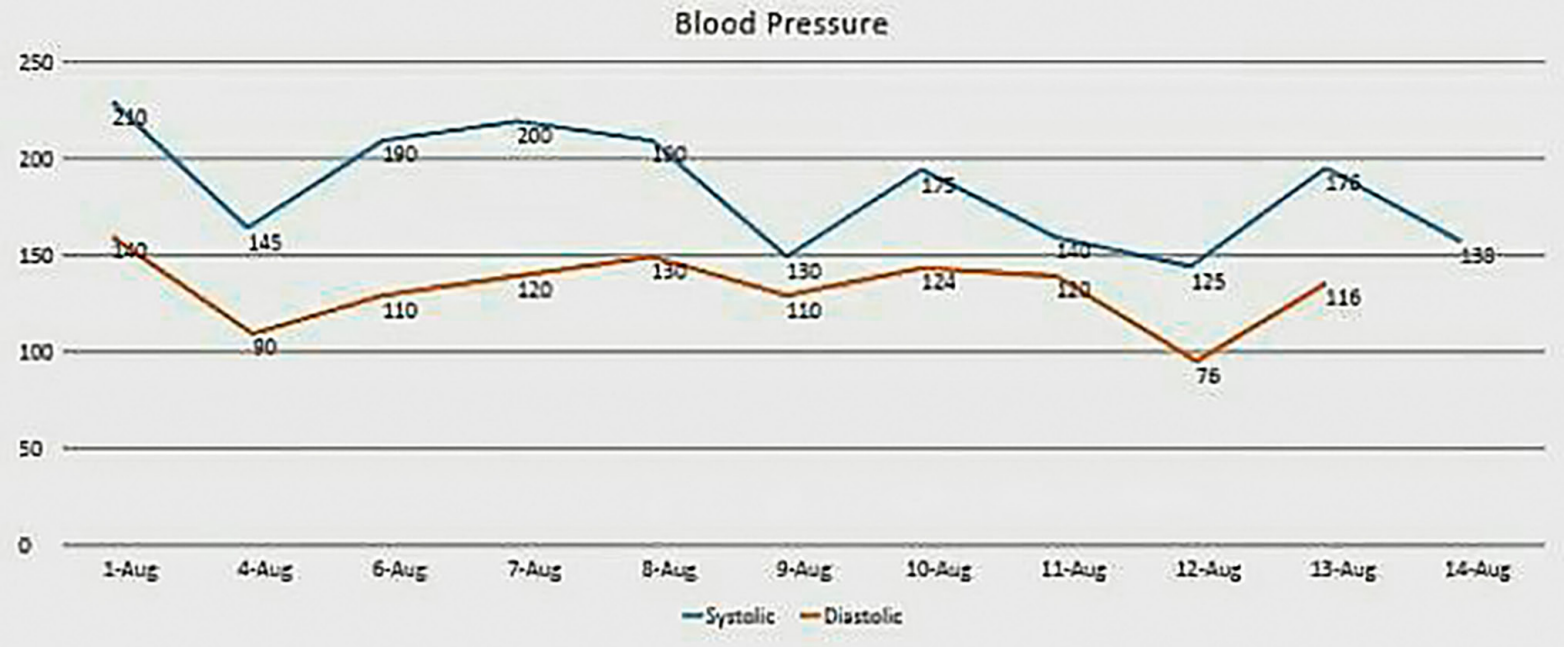
Postoperative blood pressure chart

She underwent left‐sided adrenalectomy. About 2 cm × 2 cm firm mass was seen in the left adrenal gland, which was removed (Figure 2). Histopathology confirmed the mass to be pheochromocytoma. Postoperatively, the blood pressure decreased gradually (Figure 3).

The urine markers for pheochromocytoma decreased after the operation (Table 2).

The patient was discharged on the 10th postoperative day on antihypertensive medications. She was not able to follow up in our hospital due to the long distance travel. Therefore, she was advised to follow up in the nearby hospital.

## 3 DISCUSSION

A pheochromocytoma is a catecholamine‐secreting tumor commonly arising from the adrenal medulla. It is usually benign, and unilateral but may be bilateral when associated with the familial disorder. This tumor occurs in less than 1% of all hypertensive patients.^5^ Pheochromocytoma during pregnancy is rare and the diagnosis may be challenging as it masquerades with other causes of hypertension in pregnancy.

Symptoms due to pheochromocytoma in pregnancy are the same as in nonpregnant patients. Although episodic hypertension associated with a triad of headache, palpitation, and excessive sweating is the classic triad of hypertension, one third of the cases can have sustained hypertension.^6^ Sustained hypertension indicates continuous catecholamine release and it corresponds with a high circulating level of catecholamines.^7^ In addition, 20% of patients also have proteinuria creating confusion with preeclampsia.^8^

Symptoms in pregnancy can be triggered by enlarging the uterus, uterine contraction, fetal movements, and anesthesia.^9^ As a result, patient symptoms will increase as the pregnancy progresses. Differentiating pheochromocytoma from pregnancy‐induced hypertension is challenging as most features are similar (Table 1). Comparing our case to common differentiating points, we found overlap in clinical features. Timing of the start of symptoms was important in our case as our patient was diagnosed with chronic hypertension in the 8th week of pregnancy. Therefore, physicians should keep pheochromocytoma as a differential to be ruled out in hypertensive pregnant individuals in the first trimester.

Anesthetic management of any surgical patient with pheochromocytoma is challenging especially if the tumor has not been diagnosed. In undiagnosed cases, induction of anesthesia may precipitate a hypertensive crisis. In this situation, mortality is close to 80%.^10^ Anesthetic drugs may also exacerbate the life‐threatening cardiovascular effects of catecholamines secreted by these tumors. Fortunately, such complications were not noted in our case at the time of the initial C‐section. However, the missed diagnosis may have led to serious anesthetic complications and mortality.

A biochemical test followed by imaging for localization of tumor is done in suspected cases of pheochromocytoma. 24 h urine metanephrines and catecholamines are used as the confirmatory test. However, an initial plasma‐free metanephrine test may be used when there is a high suspicion of pheochromocytoma.^11^ MRI without gadolinium is a test of choice for localizing tumors in pregnancy over CT scan due to the risk of radiation hazards.^12^ Ultrasound may be useful in the first trimester, but the sensitivity is questionable in the second and third trimesters as a gravid uterus reduces the visualization of the tumor^13^. In our case, 24 h urine metanephrines and catecholamines were followed by a CT scan for localization. As our patient was in the postpartum period, there was no risk of radiation hazard to the baby.

Surgery is the definitive treatment for pheochromocytoma. But, when it occurs during pregnancy, the timing of surgery is important. Surgery is preferred in less than 24 weeks of gestation; beyond that medical management till the term followed by adrenalectomy during C‐section is preferred.^14^ Our patient came to us after a C‐section on the fourth postpartum day. Therefore, adrenalectomy was performed. Although pheochromocytoma is associated with poor fetal outcomes, in our case, the fetus was healthy and doing well. In other case reports in the event of undiagnosed pheochromocytoma, hypertensive emergencies, acute pulmonary edema, malignant arrhythmias, myocardial ischemia or infarction, aortic dissection, cardiac failure and hemodynamic collapse, and death have been described.^15^

After definitive management of pheochromocytoma, blood pressure usually goes back to a normal level.^16^ Annual long‐term follow‐up is generally indicated as about 15% of patients can have a recurrence^17^. As our patient lived far away from our institution, a long‐term follow‐up plan was advised to her to a nearby hospital.

The authors feel that due to the rarity of pheochromocytoma in pregnancy, a multicenter study or a system of referral of cases to a single dedicated center will be required to better understand the characteristics of the disease and the incidence of missed diagnosis in Nepal.

## 4 CONCLUSION

Atypical cases of hypertension in pregnancy should be investigated early and differentiated from preeclampsia. Despite the low prevalence, pheochromocytoma in pregnancy increases feto‐maternal morbidity and mortality. Early recognition and treatment can drastically change the outcome.

## AUTHOR CONTRIBUTIONS

Swati Kumari (SK) and Ramesh Lamichhane (RL) involved in the concept of study and design. SK, RL, Saroj Chaudhary (SC), and Neha Dangol (ND) were involved in the literature review and preparation of the draft of the article. Subarna Giri (SG) and Shanta Neupane (SN) were involved in the preparation of the final article and editing. All authors individually did the final proofreading of the article before submission.

## FUNDING INFORMATION

None.

## CONFLICT OF INTEREST

The authors declare that they have no competing interests.

## ETHICAL APPROVAL

As case reports are exempt from ethical approval in our institution, our article which describes a case report does not require additional permissions from the Ethics committee.

## CONSENT

Full written informed consent was obtained from the patient for publication of her case, clinical images, and radiographic images. A copy of written consent can be made available to the editor in chief of this journal upon request.

## Data Availability

All the data generated or analyzed during this study are included in the manuscript.

## References

[1] Santos DRPD , Barbisan CC , Marcellini C , dos Santos RMVR . Pheochromocytoma and pregnancy: a case report and review. J Bras Nefrol. 2015;37(4):496‐500. 26648500 10.5935/0101-2800.20150078

[2] Biggar MA , Lennard TWJ . Systematic review of phaeochromocytoma in pregnancy. Br J Surg. 2013;100(2):182‐190. 23180595 10.1002/bjs.8976

[3] Chen H , Sippel RS , O'Dorisio MS , et al. The north American neuroendocrine tumor society consensus guideline for the diagnosis and management of neuroendocrine tumors: pheochromocytoma, paraganglioma, and medullary thyroid cancer. Pancreas. 2010;39(6):775‐783. 20664475 10.1097/MPA.0b013e3181ebb4f0 PMC3419007

[4] Ghalandarpoor‐Attar SN , Ghalandarpoor‐Attar SM , Borna S , Ghotbizadeh F . A rare presentation of pheochromocytoma in pregnancy: a case report. J Med Case Reports. 2018;12(1):37. 10.1186/s13256-017-1549-z PMC5806440 29422092

[5] Omura M , Saito J , Yamaguchi K , Kakuta Y , Nishikawa T . Prospective study on the prevalence of secondary hypertension among hypertensive patients visiting a general outpatient clinic in Japan. Hypertens Res. 2004;27(3):193‐202. 15080378 10.1291/hypres.27.193

[6] Sheps SG , Jiang NS , Klee GG . Diagnostic evaluation of pheochromocytoma [internet]. Endocrinol Metab Clin North Am. 1988;17:397‐414. Available from. doi:10.1016/s0889-8529(18)30426-2 3042392

[7] Zuber SM , Kantorovich V , Pacak K . Hypertension in pheochromocytoma: characteristics and treatment. Endocrinol Metab Clin North Am. 2011 Jun;40(2):295‐311. vii. 21565668 10.1016/j.ecl.2011.02.002 PMC3094542

[8] Keely E . Endocrine causes of hypertension in pregnancy‐‐when to start looking for zebras. Semin Perinatol. 1998;22(6):471‐484. 9880117 10.1016/s0146-0005(98)80027-x

[9] Oliva R , Angelos P , Kaplan E , Bakris G . Pheochromocytoma in pregnancy: a case series and review. Hypertension. 2010;55(3):600‐606. 20083723 10.1161/HYPERTENSIONAHA.109.147579

[10] Myklejord DJ . Undiagnosed pheochromocytoma: the anesthesiologist nightmare. Clin Med Res. 2004;2(1):59‐62. 15931336 10.3121/cmr.2.1.59 PMC1069072

[11] Vora KS , Shah VR . Diagnosis of pheochromocytoma. J Anaesthesiol Clin Pharmacol. 2012;28(2):274‐276. 22557771 10.4103/0970-9185.94931 PMC3339753

[12] Reisch N , Peczkowska M , Januszewicz A , Neumann HPH . Pheochromocytoma: presentation, diagnosis and treatment. J Hypertens. 2006;24(12):2331‐2339. 17082709 10.1097/01.hjh.0000251887.01885.54

[13] Prete A , Paragliola RM , Salvatori R , Corsello SM . Management of catecholamine‐secreting tumors IN pregnancy: a review. Endocr Pract. 2016;22(3):357‐370. 26536138 10.4158/EP151009.RA

[14] Lenders JWM . Pheochromocytoma and pregnancy: a deceptive connection. Eur J Endocrinol. 2012;166(2):143‐150. 21890650 10.1530/EJE-11-0528

[15] Desai AS , Chutkow WA , Edelman E , Economy KE , Dec GW Jr . Clinical problem‐solving. a crisis in late pregnancy. N Engl J Med. 2009;361(23):2271‐2277. 19955528 10.1056/NEJMcps0708258

[16] Falhammar H , Kjellman M , Calissendorff J . Treatment and outcomes in pheochromocytomas and paragangliomas: a study of 110 cases from a single center. Endocrine. 2018;62(3):566‐575. 30220006 10.1007/s12020-018-1734-x PMC6244895

[17] Amar L , Servais A , Gimenez‐Roqueplo AP , Zinzindohoue F , Chatellier G , Plouin PF . Year of diagnosis, features at presentation, and risk of recurrence in patients with pheochromocytoma or secreting paraganglioma. J Clin Endocrinol Metab. 2005;90(4):2110‐2116. 15644401 10.1210/jc.2004-1398

